# Synergistic effect of honeybees and wild floral visitors in promoting sweet cherry fruit set in central Chile

**DOI:** 10.1186/s40659-025-00617-2

**Published:** 2025-06-12

**Authors:** Camila B. García, Pablo Díaz-Siefer, Cecilia Smith-Ramírez, Fernanda Montero-Silva, Jaime Martínez-Harms, Maureen Murúa, Juan L. Celis-Diez

**Affiliations:** 1 Escuela de Agronomía, Pontificia Universidad Católica de Valparaíso, Quillota, Chile; 2 Centro Regional de Investigación e Innovación para la Sostenibilidad de la Agricultura y los Territorios Rurales (Ceres), Quillota, Chile; 3 Departmento de Ciencias Biológicas y Biodiversidad, Universidad de Los Lagos, Osorno, Chile; 4 Instituto de Conservación, Biodiversidad y Territorio, Universidad Austral de Chile, Valdivia, Chile; 5 Institute of Ecology and Biodiversity (IEB), Santiago , Chile; 6 Instituto de Investigaciones Agropecuarias, INIA-La Cruz, La Cruz, Chile; 7 Centro GEMA: genómica, ecología y medio ambiente, Universidad Mayor, Santiago, Chile

**Keywords:** Agroecology, *Apis mellifera*, *Bombus terrestris*, Crop pollination, Ecological intensification, Ecosystem services

## Abstract

**Background:**

Recent evidence highlights the key role of wild insects as pollinators in agroecosystems, enhancing fruit set in crops such as sweet cherry (*Prunus avium*). In Chile, the contribution of wild insects to crop yield remains poorly understood, and most farmers rely on managed *Apis mellifera* or *Bombus terrestris* for sweet cherry pollination. Here we evaluate the role of wild and managed floral visitors’ taxa in fruit sets of sweet cherry cultivated in Mediterranean-type ecosystems of central Chile.

**Methods:**

The contribution of (i) *Apis mellifera*, (ii) wild insects, and (iii) *Bombus terrestris* floral visitors were analyzed using a Linear Mixed Model with visitation rate of each group as a fixed factor and a fruit set as a response variable. Orchards were included as a random factor.

**Results:**

We recorded 24 species of floral visitors. *Apis mellifera* was the most frequent visitor, as the orchards supplemented pollination with beehives, followed by visits from wild insects and *B. terrestris*. Our results revealed that interaction between honeybees and wild insects significantly promoted higher fruit sets, while no effect of *B. terrestris* or *A. mellifera* visits alone was observed.

**Conclusions:**

We argue that wild insects contribute to the sweet cherry fruit set in the Mediterranean-type ecosystems of Chile, complementing the pollination services provided by *A. mellifera*. Our study reinforces the evidence about the importance of promoting wild floral visitors’ presence to enhance pollination and move toward more sustainable agriculture systems.

**Supplementary Information:**

The online version contains supplementary material available at 10.1186/s40659-025-00617-2.

## Background

Most flowering plants (approximately 78–94%) depend on biotic pollination, with insects being the most important pollinators [1]. Insect pollinators play a crucial role in agroecosystems, contributing to the production of 75% of the world’s crop species and accounting for 35% of global food production [2]. In Mediterranean-type ecosystems (hereafter MTE), domesticated Apidae (e.g., mainly the honeybee *Apis mellifera* Linnaeus and the buff-tailed bumble bee *Bombus terrestris* Linnaeus) have traditionally been introduced as an agronomic input to enhance crop production [3, 4]. However, despite the massive abundance of managed bees in agroecosystems, their potential impact on wild pollinator communities and disruption of plant-wild pollinator interactions [5–9], several studies have shown that a higher diversity of wild floral visitors improves pollination services and increase crop productivity [10–14]. Therefore, management strategies that promote the coexistence of crop fields with biodiversity (i.e., ecological intensification practices), may enhance the provision of ecosystem services such as pollination and biological pest control by wild insects, making them steadier [15, 16].

Chile is one of the world’s leaders in fruit exports and the largest exporter of cherries to the northern hemisphere [17]. However, the areas where cherry is cultivated overlaps with the distribution range of the sclerophyllous vegetation in the MTE of central Chile, which comprises a global biodiversity hotspot that combines extremely high endemism and threatened flora and fauna [18, 19], constituting the habitat of about 70% of Chile’s bee species [20]. Recent studies have highlighted an important role of wild insects in crop productivity in MTE of central Chile [21, 22].

The sweet cherry crop (*Prunus avium* Linnaeus) is one the leading crops cultivated in MTE of central Chile (≈ 355 thousand tons exported in 2021/2022 season). Being highly dependent on insects for pollination [2, 23], the honeybee (*Apis mellifera*), has traditionally been used to manage cherry pollination [24]. In addition, the buff-tailed bumble bee (*Bombus terrestris*), has become widely recommended by technical advisors [25, 26].

Studies in northern hemisphere farms have documented that the decline of wild insects resulted in pollination deficit in sweet cherry orchards [13, 27]. On the other hand, different studies have shown that wild bees play a crucial role in the fruit set of this crop [24, 28–30]. For example, Bakhsi et al. [31], analyzing pollination effectiveness based on the pollination probability index, PPI *sensu* Ne’eman et al. [32], reported higher PPI of wild insect visitor communities compared to honeybees in northern India. Additionally, a study in sour cherry (*Prunus cerasus* Linnaeus) revealed a higher PPI for the wild Chilean bee *Corynura cristata* Smith (Hymenoptera: Halictidae) than for honeybees [33]. Despite this information, the overall contribution of wild insects to sweet cherry fruit set cultivated in MTE of central Chile has so far not been evaluated [34]. We studied the floral visitors of sweet cherry in central Chile and evaluated the potential contribution of managed pollinators and wild insects on sweet cherry fruit set. We hypothesize that wild insects’ flower visits increase sweet cherry fruit set regardless of the visits of managed honeybees and bumble bees.

## Materials and methods

### Site study

**Fig. 1:**
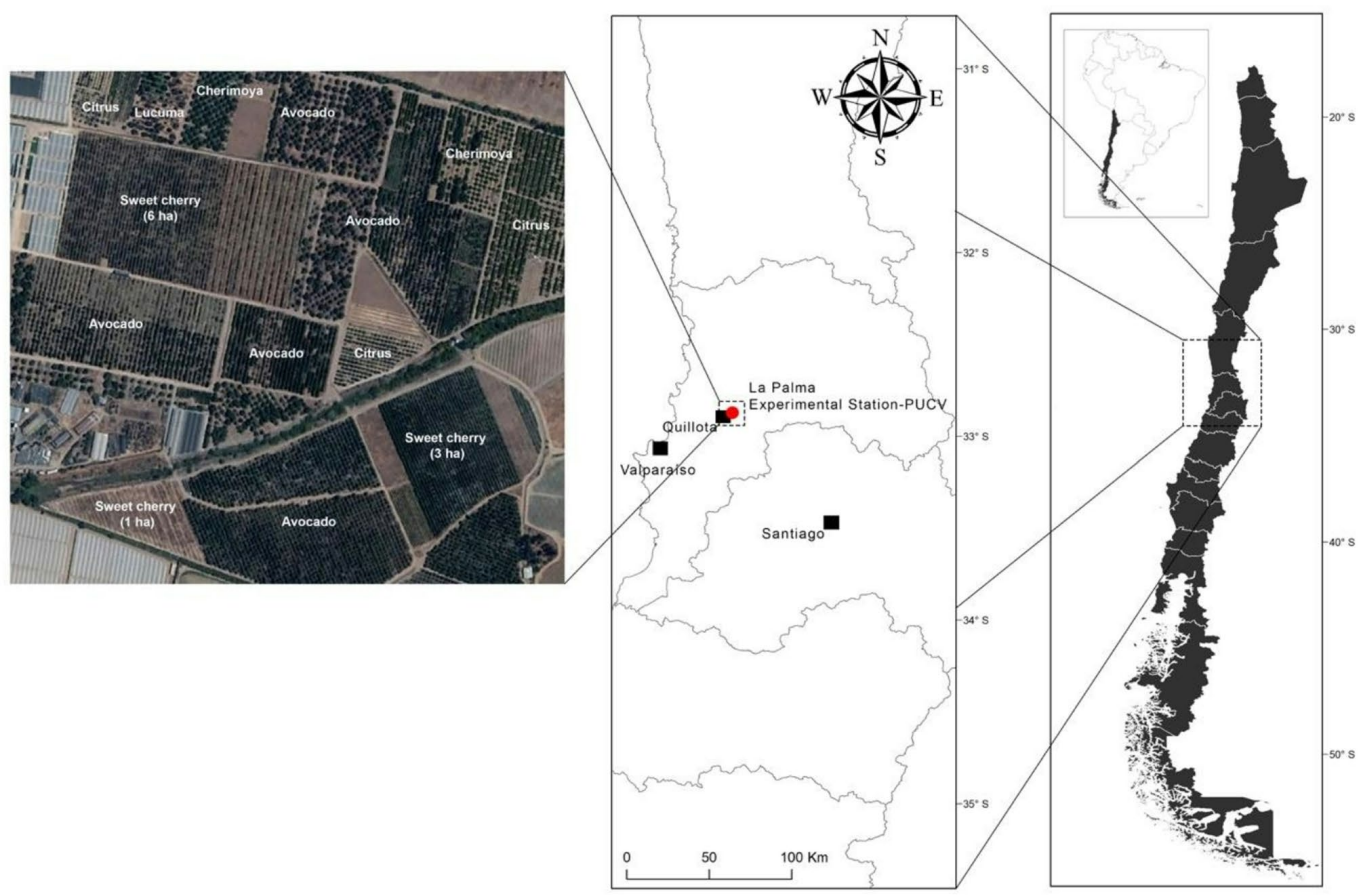
Spatial configuration of the studied orchard and surrounding fruit crops

The study was conducted in three conventionally managed sweet cherry orchards (*Prunus avium*), Brooks variety, located at La Palma Experimental Station Farm (EELP, 32° 53’S, 71° 12’W) in Quillota valley, Valparaiso, Chile. The farm belongs to the Faculty of Agricultural and Food Sciences of the Pontificia Universidad Católica de Valparaíso, Chile (Fig. 1). Sweet cherry, var. Brooks has a high dependence on pollinators to produce fruits [23].

The orchards sizes were ca. 6, 3, and 1 ha respectively, immersed in a mix of other fruit crops including avocado, citrus, cherimoya, and walnut orchards (Fig. 1). Sampled sweet cherry orchards were separated by at least 150 m and were located 800 m away from a border of native sclerophyllous vegetation remnant area (Fig. 1). In these orchards, 15 honeybee hives and five buff-tailed bumble bee colonies per hectare were used to supplement pollination.

### Sampling of insect floral visitors and fruit set

Field observations were carried out during the blooming peak of the sweet cherry crop in the Austral spring from September 3rd to 12th, 2019 (i.e., 10 days). This sampled period includes the entire flower’s lifespan, and each flower stay in anthesis between 2 and 3 days in MTE [35]. A total of 50 trees were randomly chosen from the three sampled orchards (distributed such as 26, 13, and 11 trees in the 6, 3, and 1 ha orchards, respectively; Fig. 1). Each selected tree was separated from the others by at least four rows. At each selected tree, an easily observable experimental unit consisting of a 1 m^2^ plot was defined at a minimum height of 1.5 m at the tree canopy [21]. At each experimental unit, flowers were counted, and floral visitors were recorded for 5 min by direct observations (following Holzschuh et al. [28] for sweet cherry and Díaz-Forestier et al. [36]). Each day, a total of 50 trees were observed by four trained observers. The number of trees per observer varied: some assessed 10 trees, while others observed more to reach the daily total. Sweet cherry flowers produce nectar continuously throughout the day [37]. Therefore, to account for the great diversity of floral visitors at each sampling plot, we carried out six observations per tree [38]: three in the morning (10:00 to 13:00) and three in the afternoon (15:00 to 17:00), corresponding to the warmest periods of the day. We designed this sampling schedule to maximize pollinator activity, as a previous study on crops in Mediterranean Chile found a strong correlation between temperature and bee visitation rates [39]. We excluded the 13:00–15:00 h period due to high temperatures, which can reduce pollinator activity [40]. Thus, we optimized pollinator detection while minimizing heat-related declines with the chosen sampling periods. A legitimate pollinator visit was counted when the insect contacted stigmas while feeding/foraging for nectar or pollen. A total of 1500 m. of observations were carried out in the orchards. The number of open flowers was counted at each plot at each sampling period. During sampling we recorded: (i) floral visitors’ richness (total number of insect (species/morphospecies) observed during the sampling); (ii) abundance (total number of individuals of each species/morphospecies observed during the sampling), and (iii) visitation rates (total number of insects of each species divided by the number of open flowers per plot). Based on the authors’ expertise, we identified species to the lowest taxonomic level possible in the field. When direct identification was not possible, photographs were taken and/or specimens were mist-netted for lab identification. We used taxonomic keys and scientific articles to obtain criteria for species or genus identification and consulted entomology specialists when necessary.

To assess fruit set, as a proxy of sweet cherry yield (see Eeraerts et al. [24]), we returned to the focal trees after four weeks in mid–October and counted all developed green cherries (i.e., initial fruit set) at the same previously labeled sampling plots [24]. Fruit set proportion was defined as the number of developed fruits divided by the number of initial flowers. The values of fruit set proportion were arcsine transformed prior to analysis (see Garratt et al. [41]). We quantified the initial fruit set because it is an important determinant of yield but is independent of many other factors that may induce fruit fall subsequently. For the analysis, data were averaged for the six observation periods for each tree.

Despite the presence of beehives along the study sites and the widespread occurrence of honeybees in rural areas of MTE, we classified the observed floral visitors into three groups: *Apis mellifera* and *Bombus terrestris* (both managed bees), and wild insects. It is important to note that no pollination exclusion experiments were carried out.

### Statistical analysis

We evaluated the relation between fruit set and floral visitation rates of the different groups of insects (i.e., *(A) mellifera*,* (B) terrestris*, and wild insects) using a Linear mixed model (LMM) with the arcsine transformed fruit set [41] as the response variable (similar to Holzschuh et al. [28]) and the different floral visitors as fixed factors. We included the three orchards as random factors.

Previously, we performed the Shapiro-Wilk test of normality and a Mantel test to estimate the degree of spatial autocorrelation in the response variable (i.e., honeybee, wild insects, and buff-tailed bumble bee visitation rates) and the geographic position of the sampled trees, as we are aware of the spatial limitation of our study. All statistical analyses were conducted using the statistical software R v4.2.0 [42] on Rstudio v.1.3.1093 [43]. We used the “lme4” package for the linear mixed model [44] and the “ade4” package with 9999 permutations for the Mantel test [45].

## Results

The sampled plots had 938 ± 51 (mean + ES) flowers in anthesis (mean of 973, 1164, and 902 in the 6, 3, and 1 ha orchards, respectively) and 342 ± 17 fruits (mean of 309, 330, and 361 in the 6, 3, and 1 ha orchards, respectively). We recorded a total of 1264 floral visitors from 24 species, including managed honeybees and the buff-tailed bumble bees. Floral visitors belonged to six orders, including Hymenoptera, Diptera, Coleoptera, Lepidoptera, Hemiptera, and Psocoptera (Table 1). The honeybee was the most frequent floral visitor, accounting for 93% (*n* = 2797) of the observed visits (Table 1), followed by wild insects (4.7%, *n* = 141) and the buff-tailed bumble bee (2.3%, *n* = 68).

**Table 1:** Taxonomic list of floral visitors observed in sweet cherry orchard during the 2019 flowering season. Values are accumulated floral visitor frequency and percentages (%)

Order	Family	Species	Frequency	%
Hymenoptera	Apidae	*Apis mellifera*	2797	92.99
		*Bombus terrestris*	68	2.26
	Halictidae	*Callistochlora chloris*	7	0.23
Diptera	Syrphidae	*Allograpta* sp.	30	1.00
		*Eristalis* sp.	8	0.27
		*Syrphus* sp.	11	0.37
		*Platycheirus* sp.	2	0.07
	Tipulidae	*Tipula* sp.	5	0.17
		morpho 2 Tipulidae	8	0.27
	Anthomyliidae	*Laparia sp.*	8	0.27
		Anthomyliidae morpho 2	3	0.10
	Bibionidae	*Dilophorus* sp. morpho 1	3	0.10
		*Dilophorus* sp. morpho 2	3	0.17
	Chinoromidae	Chinoromidae morpho 1	3	0.10
		Chinoromidae morpho 2	2	0.07
	Empididae	Empididae morpho 1	2	0.07
	Muscidae	*Musca domestica*	9	0.30
	Calliphoridae	Calliphoridae morpho1	4	0.13
Coleoptera	Melyridae	*Astylus trifasciatus*	11	0.37
	Coccinelidae	*Cycloneda* sp	5	0.17
Lepidoptera	Hesperiidae	*Hylephila fasciolata*	6	0.20
	Papilionidae	*Battus polydamas*	3	0.10
Hemiptera	Psyllidae	Psyllidae morpho 1	6	0.20
Psocoptera		Psocoptera morpho 1	2	0.07

Considering all the sampled plots and observation periods (*n* = 50 plots with six observations each), the honeybee was recorded in 73% ± 0.19 (mean ± SD) of them, and wild insects and the buff-tailed bumble bee in 26% ± 0.2 and 10% ± 0.22, respectively. In almost half of the studied plots (*n* = 22), only honeybees and wild insect visits were recorded, while in twelve plots, the three groups of floral visitors were recorded. In twelve plots, only honeybee visits were recorded.

Among wild insects, species from the taxonomic Order Diptera were the most frequent visitors accounting for 72% (*n* = 101) of the visits, where Syrphids were the most frequent floral visitors (Table 1; Fig. S1). Other representative taxonomic orders were Coleoptera (11.2%), Lepidoptera (6.3%), and Hymenoptera (4.9%; Table 1).

Sampled trees showed no spatial autocorrelation in the visitation rates (Mantel test = -0.05; *P* = 0.59 for honeybees; 0.04; *P* = 0.18 for wild insects; and − 0.06; *P* = 0.61 for buff-tailed bumble bee). Thus, each tree was considered as independent replicates (i.e., *n* = 50).

We found a statistically significant and positive interaction only to *A. mellifera* and wild insects visitation rates for sweet cherry fruit set (Table 2). Regarding the other groups of floral visitors, non statistical significance was found (Table 2).

**Table 2:** Results from linear mixed regression model assessing effects of insect visitation rates (i.e., *Apis mellífera* (A), wild insects (W) and *Bombus terrestris* (B)) on the Arcsine transformed fruit set (Garratt et al., 2021). An interaction model was considered because we have visits from insects from different taxonomic groups in the same sampling plots. We included the three sampled orchards as a random factor. *P* < 0.05 in bold

Fixed Factors	Fruit set (arcsin transformed)		
	*Estimates*	*std. Error*	*p*
(Intercept)	0.67	0.11	**< 0.001**
*Apis mellifera* (A)	-0.20	0.68	0.768
Wild insects (W)	-13.31	7.21	0.072
*Bombus terrestris (B)*	-16.62	27.38	0.547
A * W	114.70	42.29	**0.010**
A * B	96.17	148.29	0.520
W * B	-330.68	2933.17	0.911
A * W * B	-6874.06	14648.05	0.641
**Random Effects**			
σ^2^	0.04		
τ_00 orchard_id_	0.00		
ICC	0.05		
N _orchard_id_	3		
Observations	50		
Marginal R^2^ / Conditional R^2^	0.307 / 0.344		

## Discussion

Our results show that honeybees were the most frequent floral visitor to sweet cherry (~ 90%), with a higher frequency than reported for Europe (e.g., ~ 70%) [30, 46]. Twenty-two wild insect species were recorded visiting sweet cherry flowers (Table 1). The total number of floral visitors constitutes an interesting result and is similar to other crops studied in the region (e.g., 23 species for avocado [21]). Considering that the studied orchards were immersed in an intense agricultural landscape, surrounded by other commercial crops (Fig. 1), a lower bee diversity was expected [28, 47]. However, our results showed that despite the massive use of honeybee hives to supplement pollination, fruit set increased due to the interaction between *Apis mellifera* and wild floral visitors. Thus, our study provides new insights into the knowledge gap on sweet cherry pollination from the Southern Hemisphere, despite Chile’s global importance in cherry production [34].

Recent evidence showed changes in plant-pollinator interaction networks by the massive introduction of honeybees in Chilean MTE [21]. Despite the relatively lower visitation rate of wild insects compared with honeybees, our analyses suggest that wild insects might be playing a synergistic role with honeybees in explaining fruit set. Previous studies have highlighted a higher pollination performance of wild insects in cherry crops [27–29, 46, 48]. However, complementary pollination services between managed bees and other insects have previously been described for sweet cherries in Germany, where the presence of honeybees and Mason bees (*Osmia* spp.) synergically increased fruit set [30]. Wild floral visitors may alter *Apis mellifera* behavior, increasing their proportion of movements between tree rows and improving their pollination efficiency by enhancing the probability of more compatible pollen deposition [49, 50]. Therefore, a diverse pollinator community, as the one described in our results, may provide a more stable pollination service to sweet cherry in the MTE of central Chile.

Previous studies showed that when surrounded by intensive agricultural landscapes, cherry orchards receive fewer pollination services by wild insects, which translates into a lower fruit set [24]. The below optimum productivity of sweet cherries under such conditions would result from honeybees being unable to compensate for the pollination services provided by wild insects [13, 24]. Additionally, considering that polytunnels are widely used in Chile to minimize fruit cracking caused by spring rains and that these practices have been documented to induce pollination deficits in sweet cherry [27], promoting a higher diversity of pollinators becomes a relevant issue. In the case of our study, the closest distance to a remnant natural area was 800 m, which may explain the lower visitation rate of wild insects. A previous study in a nearby (ca. 30 km) sour cherry orchard adjacent to native vegetation found a similar number of wild floral visitors (*n* = 21) [33], but with a higher proportion of wild Hymenoptera species (*n* = 8) than in our study. The differences in wild bee visitation may be explained by the fact that wild bees are particularly sensitive to agriculturally dominated landscapes [47].

The contribution of wild insects to crop pollination has been previously reported in Chile [21, 22, 39, 51–54]. These studies highlight the importance of syrphids as key floral visitors in these crops, one of the most frequent floral visitors recorded in our study, and also important in the production of avocado and apple [21, 22, 53]. Additionally, among the other wild insects recorded, the native bee, *Callistochlora chloris* Spinola (Hymenoptera: Halictidae), has recently been pointed out as an important floral visitor for avocado, blueberry, and sour cherry crops in central Chile [33, 39, 54]. Considering that these wild species are relevant pollinators for native vegetation in natural habitats [55], enhancing landscape diversity or implementing ecological intensification practices (e.g., hedgerows or floral strips), could help increase wild insect visitation rates, and thereby sweet cherry production [13, 24, 27–29, 46, 56].

It is interesting to notice that despite the presence of managed buff-tailed bumble bees in the studied orchard, their visitation frequency was low in comparison with honeybees and wild insects. Studies carried out in the northern hemisphere, within the native range of buff-tailed bumble bee, revealed that this insect has a low pollination efficiency in sweet cherry compared to solitary bees [49]. Moreover, this exotic bumble bee has been shown to be highly invasive in Chile [57] and is considered a threat to biodiversity, given that its establishment in South America has been associated with drastic reductions in the populations of native bee species [58]. Our findings contribute to previous evidence for South America indicating that the use of commercial buff-tailed bumble bees for crop pollination is questionable and supports the recent call of the scientific community to limit its use for managing pollination [59].

Although our study design has spatial and temporal limitations and does not allow us to assess the effectiveness (i.e., pollen deposition) of floral visits among different taxonomic groups, our results provide valuable insights into the role of wild pollinators in central Chile. While some wild visitors may be more efficient pollinators of sweet cherry than others, further studies are needed to evaluate the specific contributions of different wild species. To capture the greatest diversity of floral visits, our sampling effort covered all three days of the flowers’ anthesis. Despite experimental limitations, our study reveals a relevant trend, providing new evidence on sweet cherry pollination in the Chilean Mediterranean-type ecosystem (MTE) and highlighting the role of wild insects, alongside honeybees, in enhancing pollination and crop productivity.

We conclude that promoting the presence of wild pollinators would allow moving toward more sustainable agriculture systems. Although, more studies are needed to advance in the understanding of wild pollinators efficiency in Chile’s MTE, we urge the need to increase agricultural practices and policies that promote ecosystems services across agricultural landscapes and environmentally friendly management, such as promoting higher farm heterogeneity, and conserving and restoring native vegetation remnants [16, 60].

## Electronic supplementary material

Below is the link to the electronic supplementary material.

Supplementary Material 1

## Data Availability

Data is available at the Figshare repository https://figshare.com/s/45e4d2df66673075c06e.
