# Supplementary material for: Synergistic effect of honeybees and wild floral visitors in promoting sweet cherry fruit set in central Chile
Source: Biol Res. 2025 Jun 12;58:39. doi: 10.1186/s40659-025-00617-2 (PMC12160378; doi:10.1186/s40659-025-00617-2)

**Supplementary material**

**Synergistic effect of honeybees and wild floral visitors** **in promoting sweet cherry fruit set in central Chile**

Camila B. García, Pablo Díaz-Siefer, Cecilia Smith-Ramírez^5^, Fernanda Montero, Jaime Martínez, Maureen Murúa, Juan L Celis-Diez

**Figure S1**. Wild floral visitor from the genus *Platycheirus* (Diptera: Syrphidae), observed in the fieldwork at the sweet cherry farm in the Quillota locality. Photo by C.B. García.


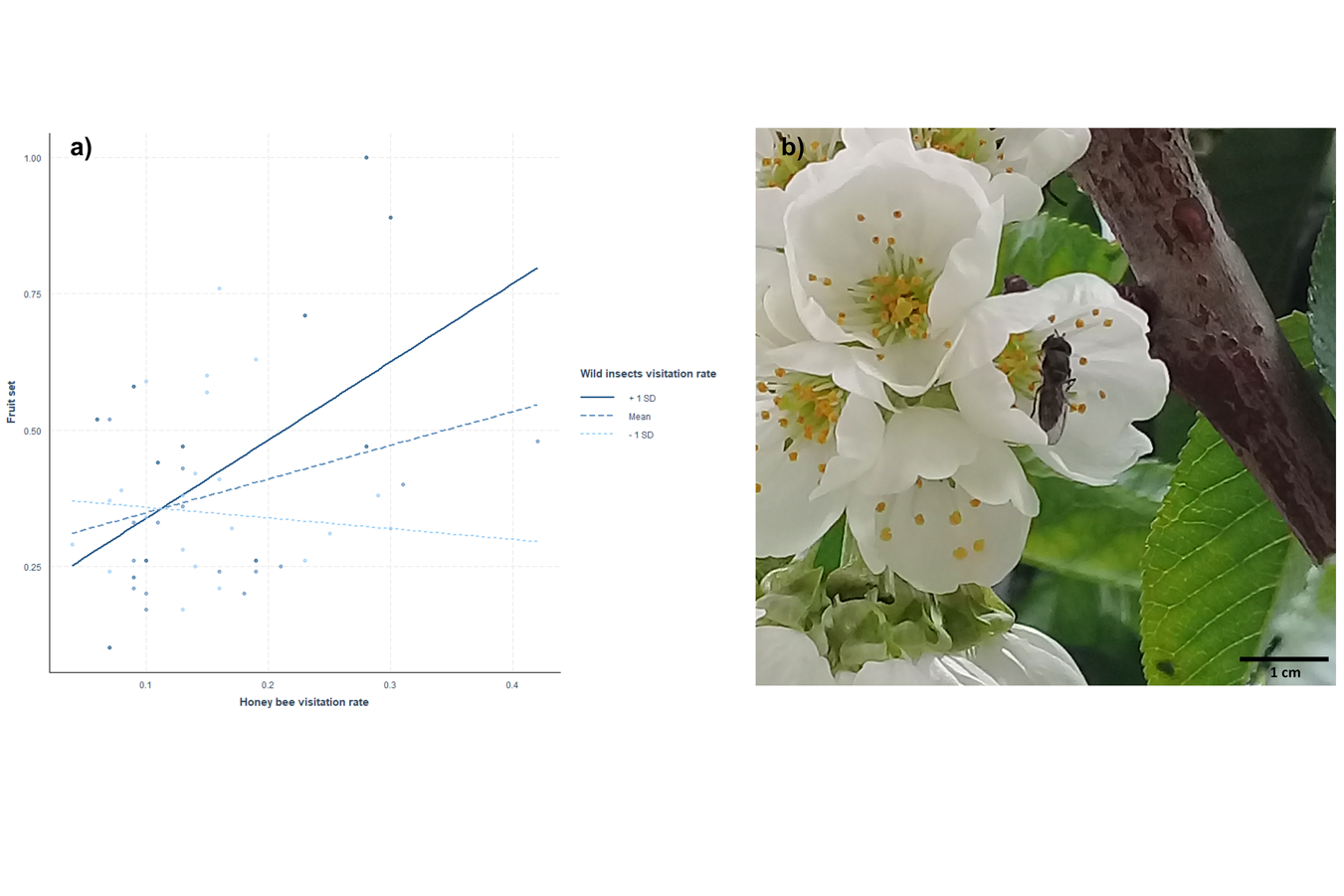

Supplement: Supplementary file 1 — Supplementary Material 1 [file 40659_2025_617_MOESM1_ESM.docx]
